# Draft Genome Sequences of *Bifidobacterium angulatum* GT102 and *Bifidobacterium adolescentis* 150: Focusing on the Genes Potentially Involved in the Gut-Brain Axis

**DOI:** 10.1128/genomeA.00709-15

**Published:** 2015-07-02

**Authors:** Marina S. Dyachkova, Ksenia M. Klimina, Alexey S. Kovtun, Natalia V. Zakharevich, Venera Z. Nezametdinova, Olga V. Averina, Valery N. Danilenko

**Affiliations:** a Vavilov Institute of General Genetics, Moscow, Russia; b Moscow Institute of Physics and Technology, Moscow, Russia

## Abstract

The draft genome sequences of *Bifidobacterium angulatum* GT102 and *Bifidobacterium adolescentis* 150 strains isolated from the human intestinal microbiota are reported. Both strains are able to produce gamma-aminobutyric acid (GABA). Detailed genomes analysis will help to understand the role of GABA in the functioning of gut-brain axis.

## GENOME ANNOUNCEMENT

*Bifidobacterium angulatum* and *Bifidobacterium adolescentis* species belong to the genus *Bifidobacterium* that represents an important commensal group of the human intestinal microbiota (1). Bifidobacteria as a part of gut microbiota are involved in numerous aspects of normal human physiology, including the functioning of the gut-brain axis (2–4). The communication between gut bacteria and the human nervous system can be provided by the ability of gut microbiota to produce neuroactive substances including gamma-aminobutyric acid (GABA) (5–7). *B. angulatum* GT102 and *B. adolescentis* 150 strains are able to synthesize and secrete GABA (8).

*B. angulatum* GT102 and *B. adolescentis* 150 strains were isolated from fecal samples of a healthy young Russian woman. The genome sequencing was carried out using a whole-genome shotgun sequencing approach performed on a Roche 454 GS Junior system (Roche, Switzerland) in the Vavilov Institute of General Genetics (Moscow, Russia). *De novo* genome assembly was performed using the GS De Novo Assembler (version 3.0; Roche). The automatic functional annotation results were obtained using the NCBI Prokaryotic Genome Annotation Pipeline (http://www.ncbi.nlm.nih.gov/genome/annotation_prok/).

A total of 105,590 reads were generated from the *B. angulatum* GT102 genome. Reads were assembled to an initial draft genome of 2,046,935 nucleotides at 34-fold coverage with 59.29% G+C content. The draft genome sequence consists of 17 contigs. The *B. angulatum* GT102 genome contains 1,520 coding sequences, 3 rRNA operons, and 56 tRNA genes. A total of 72 pseudogenes, 1 noncoding RNA (ncRNA) gene, 3 clusters of regularly interspaced short palindromic repeat (CRISPR) systems, and 14 frameshifted genes were predicted using the PGAAP.

A total of 95,067 reads were generated from the *B. adolescentis* 150 genome. Reads were assembled to an initial draft genome of 2,316,161 nucleotides at 20-fold coverage with 59.35% G+C content. The draft genome sequence consists of 37 contigs. The *B. adolescentis* 150 genome contains 1,830 coding sequences, 6 rRNA operons, and 54 tRNA genes. A total of 81 pseudogenes, 1 ncRNA gene, 2 CRISPR systems, and 20 frameshifted genes were predicted using the PGAAP. Four IS elements were found (belonging to the IS*3* and IS*256* families).

The global regulatory system genes were investigated. Genes of a type II toxin-antitoxin system of the RelBE families in the genome of *B. adolescentis* 150 were found (9). Five serine-threonine protein kinases of eukaryotic type genes (10) and two genes of the WhiB-like family (*whiB2* and *wblE*) (11) were annotated in both genomes.

The genes involved in the biosynthesis and transport of GABA were discovered in the genomes of both strains, *gadB* gene encoding glutamate decarboxylase and *gadС* gene encoding putative glutamate/gamma-aminobutyrate antiporter. The gene encoding monoamine oxidase involved in the catabolism of monoamines was found in the genomes of both strains. Other genes involved in the metabolism of neuroactive substances such as acetylcholine, dopamine, serotonin, histamine, and norepinephrine that can potentially be produced by bifidobacteria (5, 12, 13) were not detected.

### Nucleotide sequence accession numbers.

These whole-genome shotgun projects have been deposited in GenBank under the accession numbers LAHN00000000 (*B. angulatum* GT102) and LBHQ00000000 (*B. adolescentis* 150). The versions described in this paper are the first versions.
